# Twenty years of research on the DFS70/LEDGF autoantibody-autoantigen system: many lessons learned but still many questions

**DOI:** 10.1186/s13317-020-0126-4

**Published:** 2020-02-03

**Authors:** Greisha L. Ortiz-Hernandez, Evelyn S. Sanchez-Hernandez, Carlos A. Casiano

**Affiliations:** 1 Center for Health Disparities and Molecular Medicine, Loma Linda University School of Medicine, Loma Linda, CA 92350 USA; 2 Department of Basic Sciences, Loma Linda University School of Medicine, Loma Linda, USA; 3 Department of Medicine/Division of Rheumatology, Loma Linda University School of Medicine, Loma Linda, USA

**Keywords:** Antinuclear autoantibodies, Anti-DFS, Autoantigens, Autoimmunity, Cancer, Dense fine speckles, DFS70, Interacting partners, LEDGF/p75, PSIP1

## Abstract

The discovery and initial characterization 20 years ago of antinuclear autoantibodies (ANAs) presenting a dense fine speckled (DFS) nuclear pattern with strong staining of mitotic chromosomes, detected by indirect immunofluorescence assay in HEp-2 cells (HEp-2 IIFA test), has transformed our view on ANAs. Traditionally, ANAs have been considered as reporters of abnormal immunological events associated with the onset and progression of systemic autoimmune rheumatic diseases (SARD), also called ANA-associated rheumatic diseases (AARD), as well as clinical biomarkers for the differential diagnosis of these diseases. However, based on our current knowledge, it is not apparent that autoantibodies presenting the DFS IIF pattern fall into these categories. These antibodies invariably target a chromatin-associated protein designated as dense fine speckled protein of 70 kD (DFS70), also known as lens epithelium-derived growth factor protein of 75 kD (LEDGF/p75) and PC4 and SFRS1 Interacting protein 1 (PSIP1). This multi-functional protein, hereafter referred to as DFS70/LEDGF, plays important roles in the formation of transcription complexes in active chromatin, transcriptional activation of specific genes, regulation of mRNA splicing, DNA repair, and cellular survival against stress. Due to its multiple functions, it has emerged as a key protein contributing to several human pathologies, including acquired immunodeficiency syndrome (AIDS), leukemia, cancer, ocular diseases, and Rett syndrome. Unlike other ANAs, “monospecific” anti-DFS70/LEDGF autoantibodies (only detectable ANA in serum) are not associated with SARD and have been detected in healthy individuals and some patients with non-SARD inflammatory conditions. These observations have led to the hypotheses that these antibodies could be considered as negative biomarkers of SARD and might even play a protective or beneficial role. In spite of 20 years of research on this autoantibody-autoantigen system, its biological and clinical significance still remains enigmatic. Here we review the current state of knowledge of this system, focusing on the lessons learned and posing emerging questions that await further scrutiny as we continue our quest to unravel its significance and potential clinical and therapeutic utility.

## Introduction

Autoantibodies targeting macromolecules (e.g. DNA and proteins) associated with nuclear, cytoplasmic, and mitotic structures, commonly known as ANAs, are well established biomarkers for the differential diagnosis of systemic autoimmune rheumatic diseases (SARD) and tools in the molecular characterization of their target antigens [1, 2]. These autoantibodies, routinely detected by the HEp-2 IIFA test, are not necessarily restricted to SARD since they have been reported, albeit often at relatively lower frequencies and titers, in patients with cancer and diverse inflammatory conditions [1, 2]. Given the growing number of discovered ANAs, with their distinctive IIF patterns in HEp-2 cells, the International Consensus on ANA Patterns (ICAP) initiative (www.anapatterns.org) has recently reached consensus on the nomenclature, definition, and clinical relevance of 29 IIF ANA patterns, which are ascribed a code from AC-1 to AC-29 [3]. AC-2 defines the DFS IIF pattern as having three main features: (1) fine speckles distributed throughout the interphase nucleus with characteristic heterogeneity in their size, brightness and distribution; (2) denser and looser areas of speckles throughout the interphase nucleus; and (3) strong speckled pattern in the metaphase plate with some coarse speckles standing out.

The DFS IIF pattern is produced by autoantibodies to a nuclear, chromatin-associated protein of approximately 70 kD most commonly known as DFS70 or LEDGF/p75. Given the longstanding and widespread use of both names to refer to the same protein in the fields of autoimmunity, cancer, HIV/AIDS, and ocular diseases, we will use the term DFS70/LEDGF throughout this review. Although autoantibodies to DFS70/LEDGF were initially associated with atopic diseases and other miscellaneous inflammatory conditions, recent studies with more specific and sensitive antibody detection tests have challenged some of the initial observations while at the same time failing to provide a definite answer to the question we posed to the field in 2004: “what exactly are these autoantibodies trying to tell us?” [4]. In this review we discuss historical and current perspectives concerning our understanding of the enigmatic DFS70/LEDGF autoantibody-autoantigen system, and identify emerging questions that may guide our efforts to unearth its clinical and biological significance.

## Historical perspectives on DFS70/LEDGF biology: what we know

### Discovery and initial characterization

**Fig. 1:**
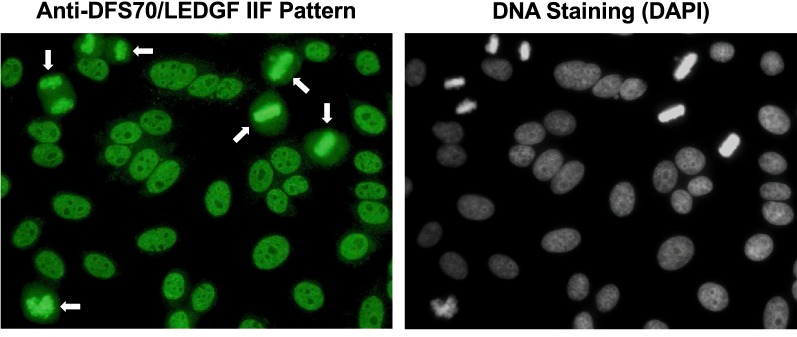
Representative dense fine speckles (DFS) pattern visualized by indirect immunofluorescence assay (IIFA) in HEp-2 cell slides using a monospecific human anti-DFS70/LEDGF serum. Arrows point to the distinctive bright staining of mitotic chromosomes

Although the initial report of the DFS IIF pattern was published in 1994 by the group of Eng M. Tan at The Scripps Research Institute, it was not until the late 1990s when autoantibodies producing this pattern were first characterized and the early glimpses into the structure and function of their target antigen began to surface [5, 6]. In their seminal paper published in 2000, Ochs et al. used serum autoantibodies presenting the DFS IIF pattern from patients with diverse atopic conditions to clone, sequence, and purify the target antigen, calling it DFS70 based both on its nuclear IIF pattern (Fig. 1) and migration around the 70 kD region in immunoblots [6]. They also noted that the entire DFS70 sequence (aa 1–530) corresponded to that of transcription co-activator p75, whereas its amino (N)-terminal region (aa 1–326) corresponded to a short splice variant of this protein, called p52, that was not recognized by the autoantibodies [6, 7]. This finding strongly suggested that the autoepitope resided in the carboxyl (C)-terminal region. These two transcriptional co-activators had been reported in 1998 by Ge et al. [7] to co-purify with the transcription co-activator PC4 and other components of the RNA polymerase II complex, and to play a role in general transcription. Ochs et al. also demonstrated by immunoelectron microscopy that the DFS70 protein was localized in interphase chromatin, concentrated over areas of condensed chromatin and also in perinucleolar chromatin, excluding the nucleoli, as well as in condensed chromosomes during metaphase and anaphase [6]. These initial studies revealed three important features of this protein: association with chromatin, component of the RNA pol II transcription complex, and C-terminal region as the target of the autoantibodies.

Working independently and contemporaneously, the group of Toshimichi Shinohara at Harvard Medical School used autoantibodies from a patient with age-related cataract to isolate a clone from a human lens epithelium cell (LEC) cDNA library encoding a protein identical to transcription co-activator p75 and DFS70 [8–10]. In several initial studies published by this group between 1999 and 2004, this protein was reported to be located in the nucleus and secreted by cultured LECs, and found to exert pro-survival effects when overexpressed or added to LECs, retinal cells, and other cell types cultured in the presence of environmental stressors such as oxidative stress, heat, and UV irradiation [8–20]. Given its protective effects, apparent secretion from cultured cells, and significant sequence homology with members of the hepatoma growth factor (HDGF) family, the protein was considered a growth factor for LECs, hence the designation LEDGF/p75 [8–10]. While subsequent studies would demonstrate that this protein is neither lens epithelium-specific nor a growth factor, the name LEDGF/p75 was widely adopted by the scientific community outside the field of autoimmunity. Singh et al. also confirmed the previous observation by Ge et al. [7] that this protein has a short alternative splice variant (p52) and demonstrated that the gene encoding both spliced forms, currently known as *Psip1*, resides in chromosome 9p22.2 [21]. Studies from this group, our group, and others have established DFS70/LEDGF as a stress activated transcription co-activator that upregulates the expression of anti-oxidant, stress response and cancer-associated genes in various cell types, particularly ocular and tumor cells [8, 11–20, 22–31]. Its role in cellular stress protection in the context of ocular diseases has been reviewed recently [32]. Table 1 provides an updated list of genes known to be upregulated by this protein.

**Table 1:** Genes regulated by DFS70/LEDGF

Gene	Function	References
ADH and ALDH	Cellular detoxification and conversion of vitamin A to retinoic acid	[20]
ALB	Antioxidant activity through multiple-binding sites and capacity to trap radicals	[26]
AOP2/PRDX6	Peroxiredoxin involved in cellular redox regulation and protection against DNA damage by reactive oxygen species	[13]
Cell cycle genes	Cell cycle progression; knockdown of DFS70/LEDGF led to reduced levels of several cell cycle and cancer-related genes in breast cancer cells	[86]
CYGB	Reactive oxygen species scavenger activated by hypoxic and oxidative conditions	[26]
FBXO10	Ubiquitin E3 ligase associated with breast cancer susceptibility	[153]
ERp57	Disulfide isomerase involved in cellular protection against cell death induced by hydrogen peroxide	[31]
HOX genes	Transcription factors involved in development and cancer that are regulated by the complex between MLL, Menin, and DFS70/LEDGF	[75, 90, 154]
HSP27	Chaperone involved in inhibition of apoptosis and stress responses	[15, 27, 30]
IL-6	Inflammatory cytokine	[28, 29]
INV	Marker of differentiation in keratinocytes; involved in keratinization	[18]
PIP3-E/IPCEF-1	Oxygen carrier involved in peroxidase activity and translocation of cytohesins to the plasma membrane	[26]
p21	Cell cycle regulator involved in DNA damage response	[155]
SOD3	Antioxidant enzyme involved in the conversion of superoxide radicals into hydrogen peroxide and oxygen	[26]
TPO	Involved in the oxidation of iodide to iodine for the synthesis of thyroid hormone	[26]
VEGF-C	Involved in tumor lymphangiogenesis and endothelial cell growth	[24, 25]
αB-crystallin	Molecular chaperone that prevents protein aggregation under stress and contributes to lens function	[23]
γGCS-HS	Antioxidant defense enzyme upregulated by tumor necrosis factor alpha via DFS70/LEDGF	[22]

### Structural/functional domains and interacting partners

**Fig. 2:**
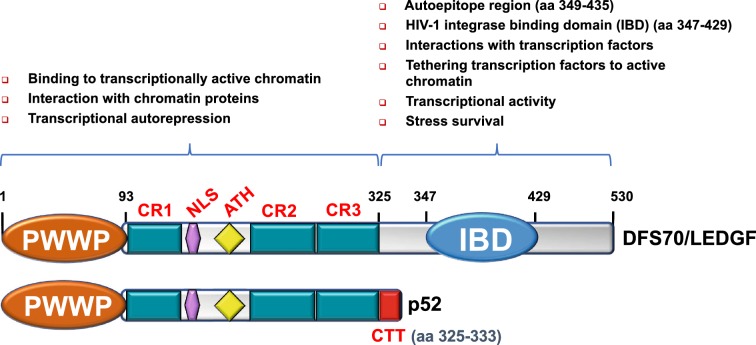
Domain structure and functions of DFS70/LEDGF and its splice variant p52. The two variants share a common amino (N)-terminal region (amino acids 1–325) comprised by a PWWP domain, a nuclear localization signal (NLS), two AT-hook DNA binding domains, and three charged regions (CR). The carboxyl (C)-terminal region of DFS70/LEDGF (amino acids 326–530) is absent in p52 and contains the HIV integrase binding domain (IBD), which overlaps with the autoepitope region recognized by the anti-DFS autoantibodies. The extreme C-terminal region of p52 contains a short intron-derived sequence (amino acids 325–333) not present in DFS70/LEDGF designated carboxy-terminal tail (CTT). The known functions of the N- and C-terminal regions are listed

The HDGF family of chromatin binding proteins, which in addition to DFS70/LEDGF also includes HDGF, HRP2/HDGF2 (HDGF-related protein 2), HRP-3, and HDGF-L1, has been implicated in promoting cancer cell proliferation and survival [33–35]. These proteins share significant sequence homology, particularly in the N-terminal region, which contains a 100 amino acid stretch called the HATH (homologous to the amino terminus of HDGF) domain. A four-residue PWWP motif (proline-tryptophan-tryptophan-proline; amino acids 19–22 in DFS70/LEDGF) is a key element of the HATH domain, hence this domain is more commonly known as the PWWP domain. In the primary structure of DFS70/LEDGF, the PWWP domain (residues 1–96) is followed by a nuclear localization signal (NLS) that is critical for interaction with nuclear import elements, two AT hook (ATH) motifs, and a supercoiled recognition domain (SRD) with three charged regions (CR1-3) [36–38]. These structural elements define the DNA binding region of both DFS70/LEDGF and its p52 alternative splice variant and are critical for the recognition of active chromatin sites (Fig. 2).

**Fig. 3:**
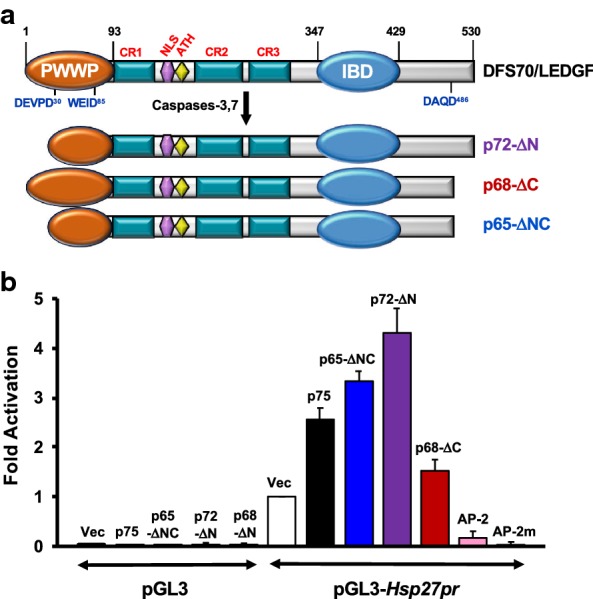
Apoptotic cleavage of DFS70/LEDGF. **a** Early during apoptosis caspases-3 and -7 cleave DFS70/LEDGF at specific aspartic acids (D30 and D486) to generate fragments p72 (truncated PWWP) and p68 (deletion of extreme C-terminal region). These fragments are subsequently cleaved to generate p65, which lacks a portion of the PWWP domain. **b** Caspase-mediated cleavage of DFS70/LEDGF influenced its ability to transactivate the *Hsp27* gene promoter (*Hsp27pr*). U2OS cells were transiently transfected with luciferase (luc) reporter plasmids empty pGL3 vector control, pGL3-*Hsp27pr*-luc, empty pcDNA3.1 + vector control (Vec), or effector plasmids encoding p75, p65, p72, p68, or irrelevant transcription factors AP-2 and AP-2 mutant as negative controls. At 48 h post-transfection, luciferase activity was measured and fold activation of promoter activity was calculated. The highest fold activation was produced by p72, which has a deletion of the N-terminal residues 1–30, consistent with autorepression activity residing in the PWWP domain. The lowest activation was produced by p68, which has deletions in both the N- and C-terminal regions, including complete removal of the PWWP domain. Results are representative from 3 independent experiments performed in triplicates

Our group’s early observation in 2001 that DFS70/LEDGF is cleaved into multiple fragments during apoptosis led us to further investigate its role in cell death and survival decisions [39]. Using various cancer cells types and apoptosis-inducing stimuli, we established that during apoptosis, caspases-3 and -7 cleave DFS70/LEDGF at specific sites, resulting in the deletion of portions of the PWWP domain and the C-terminal region [36]. These events lead to the generation of several cleavage fragments of the protein, including a prominent p65-ΔNC product that lacked pro-survival activity in cells growing under starvation stress conditions and behave as a dominant-negative protein (Fig. 3a). Notably, caspase-mediated disruption of the C-terminal region was sufficient to abrogate the stress-survival functions of DFS70/LEDGF [36]. In subsequent studies, we showed that the short splice variant p52, which lacks the C-terminal region, does not promote cell survival but rather induces apoptosis when ectopically overexpressed in cancer cells, which explained our inability to obtain cell clones stably overexpressing this protein [40]. Interestingly, p52 is cleaved by caspases-3 and -7 during apoptosis to generate a p38 fragment that also behaves as a dominant-negative, interfering with the ability of DFS70/LEDGF to transactivate the *Hsp27* promoter region in luciferase reporter assays [40]. These studies suggested an important role for the C-terminal region of DFS70/LEDGF in its pro-survival functions.

In contemporary studies, Singh et al. [41] provided evidence that both the N- and C-terminal regions of DFS70/LEDGF contribute to its stress survival activity, with the N-terminal portion responsible for DNA binding and the C-terminal region functioning as a transcriptional activation domain. Interestingly, these investigators showed that removal of the N-terminal region of DFS70/LEDGF (residues 1–187) increased the transcriptional activity of the protein in luciferase reporter assays, suggesting an auto-transcriptional repression role for this region. This repression activity is likely to reside in the PWWP domain since caspase-mediated deletion of a portion of this domain (residues 1–30) generates a p72-ΔN fragment with increased *Hsp27* promoter transactivation activity in reporter assays (Fig. 3b).

Between 2003 and 2004 the groups of Zeger Debyser (Leuven), Alan Engelman (Dana Farber), and Eric Poeschla (Mayo Clinic), reported independently that DFS70/LEDGF interacts with the human immunodeficiency virus 1 integrase (HIV-IN) and serves as a tethering factor to facilitate viral DNA integration into host chromatin. This seminal finding paved the way for numerous studies that not only elucidated the role of this transcriptional co-activator in HIV-1 integration but stimulated research into its basic biology (reviewed in [42–45]). For instance, these studies have revealed that the DFS70/LEDGF PWWP domain facilitates the recognition of di- or tri-methylated lysine 36 in histone H3 (H3K36me2/3), which serves as a marker of actively transcribed genes [46, 47]. It was recently established that the ability to bind H3K36me2/3 allows DFS70/LEDGF and the hepatoma derived growth factor protein HRP2/HDGF2 to work in concert to enable RNA pol II to overcome nucleosome-induced barrier to transcription observed in differentiated cells that no longer express the FACT (facilitates chromatin transcription) protein complex [48]. This property also allows DFS70/LEDGF to tether its interacting partners, including HIV-IN, to transcriptionally active sites in the chromatin [45, 46].

HIV-IN interacts with a C-terminal domain structure in DFS70/LEDGF, designated the integrase binding domain (IBD), that is involved in the efficient integration and replication of HIV-1 into host chromatin [49]. Studies with cells depleted of DFS70/LEDGF (knockdown or knockout) have provided compelling evidence for a critical role of this protein, particularly its IBD region, in HIV-1 integration. For instance, its transient and stable knockdown via RNA interference (siRNA or shRNA) resulted in a robust reduction of HIV-1 replication [50, 51]. Whole-gene DFS70/LEDGF deletion or deletion of the IBD by transcription activator-like nucleases (TALEN) also resulted in inhibition of HIV integration, severely impairing the spreading of viral replication [52]. In addition, knockout of the DFS70/LEDGF IBD exons through homologous recombination resulted in laboratory HIV-1 strains with severe replication delay and replication-defective clinical HIV-1 isolates [53]. Targeted editing of the *Psip1* locus encoding DFS70/LEDGF using the CRISPR technology, in this case used to mutate aspartic acid residue 366 within the IBD, successfully disrupted interaction with HIV-IN and resulted in decreased integration deficiency and HIV-1 replication [51]. Interestingly, like DFS70/LEDGF, the HRP2/HDGF2 protein also harbors a PWWP domain in its N-terminus and an IBD in its C-terminus, which allows it to maintain residual HIV-1 integration in cells depleted of DFS70/LEDGF [54]. Although DFS70/LEDGF and HRP2/HDGF2 share common domains and facilitate both RNA pol II transcription and HIV-1 integration, a direct interaction between these two proteins has not been established yet.

In addition to its chromatin binding properties, the PWWP domain also serves as a site for protein–protein interactions, as evidenced by our previous report that in prostate cancer (PCa) cells this domain facilitates the direct interaction between DFS70/LEDGF and the methyl CpG binding protein MeCP2, which may function as a transcriptional repressor or activator depending on the context [30]. The PWWP domain of DFS70/LEDGF also interacts with other chromatin-associated proteins, including the transcription co-activator TOX4, the DNA-repair associated protein CtIP, and the mRNA splicing factor NOVA1 [55, 56]. Interestingly, this domain was recently implicated indirectly in interactions with several mRNA splicing factors as a mechanism to target HIV-1 integration to highly spliced genes [57]. In addition, RNA sequencing analysis of HEK293T cells lacking DFS70/LEDGF or the IBD (via TALEN knockout) revealed significant changes in the splicing pattern of over 5000 genes, suggesting an important role for this protein in modulating alternative splicing [57]. Consistent with these observations, mutations in the *Mecp2* gene, which cause Rett syndrome (RTT), a severe neurodevelopmental disorder that predominantly affects girls, not only disrupted the interaction between DFS70/LEDGF and MeCP2 but also altered mRNA splicing in a mouse model [58]. Interestingly, a proteomic analysis revealed that both proteins have decreased expression in glaucomatous retina, implicating them in retinal protection, which would be consistent with the stress protective role ascribed to DFS70/LEDGF in retinal and other ocular cells [59].

**Fig. 4:**
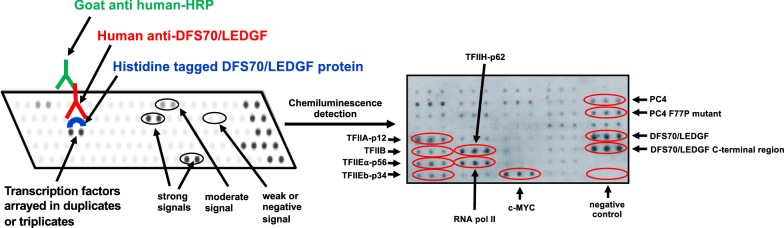
Detection of DFS70/LEDGF interacting partners using protein arrays. Commercially available transcription factor arrays were used in early studies to identify candidate interacting transcription factors of DFS70/LEDGF. Purified histidine tagged-DFS70/LEDGF was incubated with transcription factors spotted on membranes. Protein interactions were then detected with human anti-DFS70/LEDGF autoantibody, horseradish peroxidase-labeled anti-human secondary antibody, followed by exposure to chemiluminescence reagents. A representative image of a transcription factor array membrane showing positive interactions with components of the RNA pol II complex (TFII) as well as c-Myc and PC4. This approach was used for the initial detection of the interaction of DFS70/LEDGF with MeCP2 [30]

As mentioned above, the C-terminal region of DFS70/LEDGF (residues 326–530), missing in the p52 variant, contains the IBD (residues 347–429) (Fig. 2). The IBD serves as a hub for protein–protein interactions that, together with the PWWP domain, facilitates the tethering of HIV-IN and transcription factors to RNA pol II complexes at transcriptionally active sites [42–46, 49–52]. In addition to HIV-IN, a number of interacting partners of DFS70/LEDGF that bind to the IBD have been identified (Table 2). These include the mixed leukemia lineage histone lysine methyl transferase protein MLL and its binding partner Menin (MEN1), the chromatin remodeling protein PogZ, the transcription factor and c-MYC interacting protein JPO2 (also known as cell division cycle associated 7 like, CDCA7L), the transcription elongation factor and RNA pol II interacting protein IWS1, and the DNA replication associated kinase CDC7/ASK (reviewed in Refs. [45, 46]). As mentioned above, the RNA pol II transcription co-activator PC4 and several splicing factors have also been reported to interact with DFS70/LEDGF but their specific interaction with the PWWP or IBD domains is yet to be conclusively established [7, 57]. Using a transcription factor protein–protein interaction array, we detected moderate to strong protein–protein interaction signals between DFS70/LEDGF, transcription factor PC4, and RNA pol II subunits, consistent with the previous report that this protein co-purifies with these proteins (Fig. 4) [7].

**Table 2:** Known interacting partners of DFS70/LEDGF

Name	Apparent molecular weight (kD) in immunoblots	Cellular functions in the context of interaction with DFS70/LEDGF	Interacting domain	References
CDC7/ASK	65–70	Subunit and activator of S-phase kinase ASK involved in DNA replication, repair, and recombination; phosphorylates DFS70/LEDGF which in turn stimulates the kinase enzymatic activity	IBD	[45, 61]
CtIP	120–130	Endonuclease that interacts with DFS70/LEDGF to facilitate repair of DNA double strand breaks through homologous recombination	PWWP	[55]
JPO2/CDCA7L	52	Transcription factor that binds to c-Myc, mediates its transforming effect in medulloblastoma cells, and brings it in close proximity to DFS70/LEDGF in transcription complexes	IBD	[45, 46, 61, 88]
IWS1	92	Chromatin remodeler and component of RNA pol II complex; interacting partner of Spt6 transcription elongator that modulates histone methylation and production of mature mRNA transcripts; associated with Spt6 and DFS70/LEDGF for post-integration silencing of HIV-1 gene expression in HIV latency	IBD	[45, 46, 61]
Menin	68–70	Positively regulates *Hox* gene expression; required for MLL-fusion protein mediated leukemic transformation through ternary complex with MLL1 and DFS70/LEDGF	IBD	[75]
MLL	> 400	Plays key role in early development, hematopoiesis, and leukemogenesis; involved in transcriptional activation of *Hox* genes and cancer-associated genes through complex with Menin and DFS70/LEDGF	IBD	[45, 46, 61, 75]
MeCP2	55–70	Methylation-associated transcriptional modulator that interacts with DFS70/LEDGF to influence its transcriptional activity	PWWP	[30]
PC4	75	Transcription co-activator component of RNA pol II complex that interacts with both DFS70/LEDGF and its p52 splice variant during general transcription	Likely PWWP	[7]
PogZ	155	Transposase that regulates chromatin remodeling and is required for proper chromosome segregation during mitosis; its DDE endonuclease domain, necessary for efficient DNA transposition, binds DFS70/LEDGF	IBD	[45, 46]
TOX4	65–75	Transcription factor involved in transcription activation and DNA repair in complex with DFS70/LEDGF	PWWP	[56]
NOVA1	50–55	Alternative mRNA splicing cofactor that interacts with DFS70/LEDGFp75 to link RNA pol II transcription with Mrna processing	PWWP	[56]
RNA pol II	250	Polymerase that catalyzes the transcription of DNA to mRNA	Likely PWWP	[7, 86]
SRSF1 and multiple splicing factors	Vary	SRSF1 is one of a plethora of mRNA splicing factors that bind to both DFS70/LEDGF and its splice variant p52, suggesting involvement of PWWP domain; DDX5 and SNRPA splicing factors bind only to DFS70/LEDGF, suggesting involvement of IBD	Likely both PWWP or IBD	[57]
SSRP1	94	Structure specific recognition protein 1 component of the chromatin remodeler FACT (facilitates transcription complex)	PWWP	[156]

**Fig. 5:**
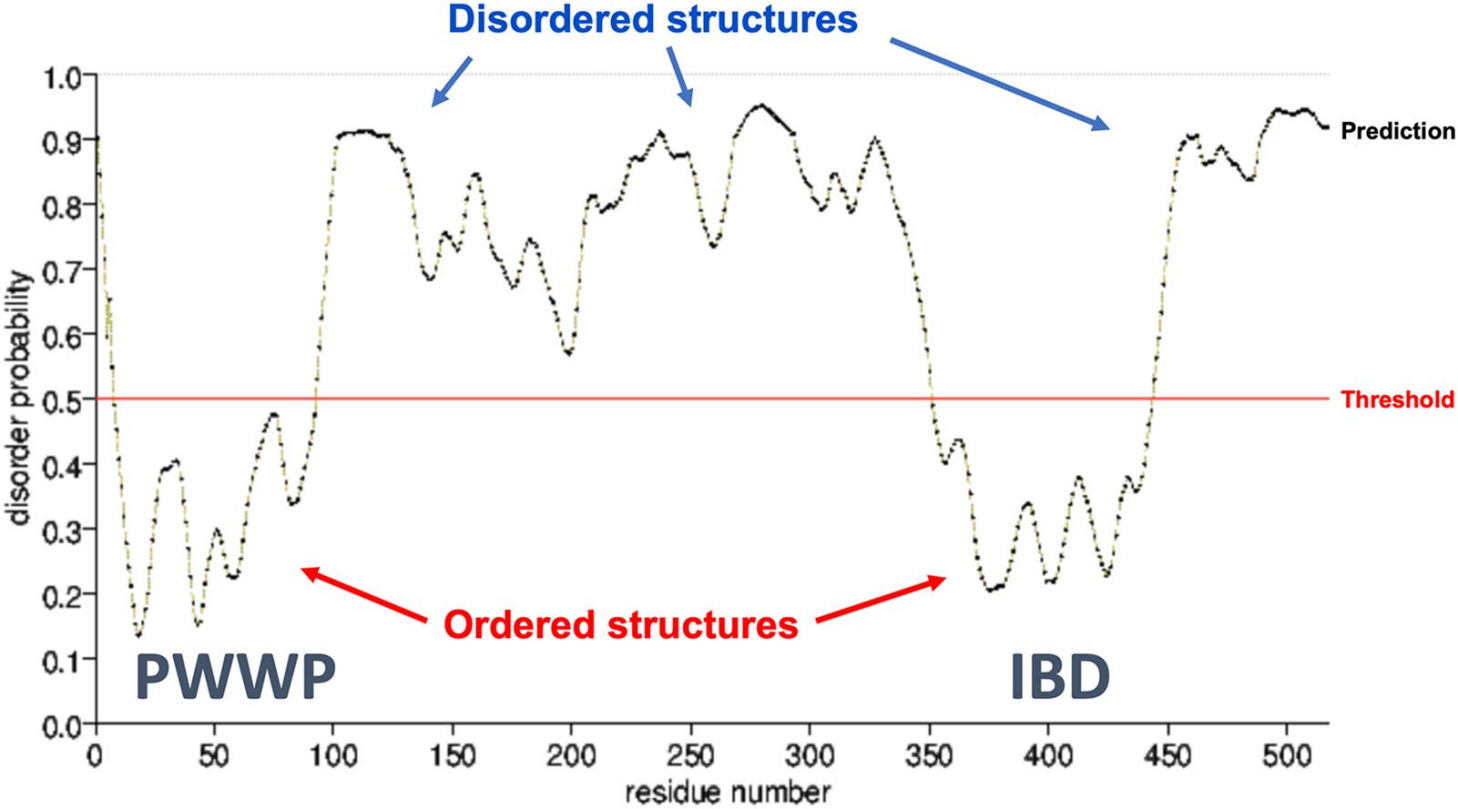
DFS70/LEDGF is an intrinsically disordered protein. The plot shows the probability of intrinsically disordered structures (IDR) in this protein. Note that the PWWP and IBD domains show low probability of disorder, with the central and extreme C-terminal regions of the protein showing high probability. Data was acquired from the PrDOS protein disorder prediction server

The interactions between DFS70/LEDGF and its partners at the IBD appear to be stabilized by intrinsically disordered regions (IDRs). For instance, a disordered IBD-binding short linear motif (IBM) was recently identified in several DFS70/LEDGF interacting partners, with their affinity for IBD binding regulated by phosphorylation of the IBM [45, 60, 61]. DFS70/LEDGF itself is also an IDR protein, having only two domains that can form stable 3D structures, the PWWP and IBD, which are separated by an IDR (residues 100–345) that lacks well-defined tertiary structure (Fig. 5). The IBD crystal structure reveals four long α-helices arranged as a helical bundle, with a fifth short helix linking two of the other α chains [62]. IDRs typically confer conformational flexibility to transcription factors, allowing them to engage in multiple transient protein–protein and protein-nucleic acid interactions that facilitate molecular events associated with transcription, DNA repair, mRNA splicing, and signal transduction [63–65]. This may explain the plasticity of DFS70/LEDGF to engage in multiple interactions with proteins (through both the PWWP and the IBD domains) and DNA to modulate transcription, DNA repair, and mRNA splicing [45, 46, 55–57]. Because of their role in various disease processes by virtue of their multiple interactions, proteins with intrinsically disordered proteins, including DFS70/LEDGF, are emerging as attractive therapeutic targets in different disease contexts [66, 67].

It should be noted that most nuclear autoantigens targeted by autoantibodies in SARD contain IDRs, which make these antigens more susceptible to proteolysis, decrease their affinity for MHC II, and diminish their representation as T cell epitopes during development of immune tolerance, consequently increasing their likelihood of becoming targets of autoantibody and T cell responses [68]. As mentioned above, we have demonstrated that DFS70/LEDGF is highly susceptible to caspase-mediated proteolysis during apoptosis, which generates several fragments of this protein that can still be recognized by human anti-DFS70/LEDGF autoantibodies [36]. Notably, these fragments retain an intact IBD, which overlaps almost perfectly with the autoepitope region recognized by these autoantibodies (residues 349–435, Fig. 3a) [36, 69]. While we have proposed previously that the apoptotic cleavage fragments of DFS70/LEDGF may trigger an autoantibody response to the IBD under inflammatory conditions [70], this still remains to be demonstrated experimentally.

### Oncoprotein functions

In addition to its roles in generating autoantibody responses, HIV-1 integration, and protecting ocular cells against stress, DFS70/LEDGF has emerged in recent years as an oncoprotein relevant to multiple cancer types. Early studies focused on its role in hematological malignancies based on observations from several groups of its involvement in fusions with the NUP98 protein generated by chromosomal translocations in patients with acute and chronic myeloid leukemia [71–73]. These chimeric proteins have NUP58 FxFG repeats fused in frame with the C-terminal IBD of DFS70/LEDGF, producing a protein that is still capable of binding DNA and tethering transcription factors to RNA pol II complexes while likely acquiring enhanced transcriptional activity due to the deletion of its auto-repressive PWWP domain. Consistent with these observations, Huang et al. [74] reported that DFS70/LEDGF has increased expression in blasts from chemotherapy resistant human acute myeloid leukemia, and that overexpression of this protein in cultured leukemic cells protected against drug-induced cell death. Subsequent studies revealed that DFS70/LEDGF critically associates with the MLL/Menin transcription complex to drive the expression of *Hox* genes and oncogenic transformation in leukemias caused by the *MLL* gene [75]. More recently, El Ashkar et al. [76] demonstrated that conditional knockout of the *Psip1* gene (which encodes DFS70/LEDGF) from blood cells in a mouse model was dispensable for normal hematopoiesis but critical for MLL-mediated leukemogenesis. Consistent with this, leukemia cells expressing MLL and overexpressing DFS70/LEDGF IBD mutants were defective for MLL interactions and displayed decreased clonogenic growth [77]. These studies have catapulted DFS70/LEDGF into the limelight of new candidate therapeutic targets for leukemia [67, 78].

DFS70/LEDGF also acts as an oncoprotein in solid tumors. Our detection of autoantibodies against DFS70/LEDGF in a subset of PCa patients in 2005 led us to examine its expression in human prostate tumors, which revealed for the first time its overexpression in solid tumors [79]. In subsequent studies, we examined DFS70/LEDGF expression in multiple cancer types, and observed significantly increased expression in prostate, colon, breast, and thyroid tumors [80]. Our group also demonstrated that ectopic overexpression of DFS70/LEDGF in PCa cells is associated with upregulation of specific stress and antioxidant proteins as well as resistance to non-apoptotic cell death induced by chemotherapy and oxidative-stress [26, 30, 31, 81]. More recent studies from our laboratory demonstrated that PCa cells selected for chemotherapy resistance activate a cancer stem cell transcriptomic program that is associated with upregulation of DFS70/LEDGF and other related proteins, and that knockdown of this protein via siRNA in these cells re-sensitizes them to taxane-based chemotherapy [82, 83]. Consistent with these results, several other groups have provided evidence that DFS70/LEDGF is also overexpressed in other cancer types, and various studies with ectopic overexpression and siRNA-mediated knockdown have shown that this protein promotes features of tumor aggressiveness, including cell proliferation, migration, invasion, clonogenicity, tumor growth, angiogenesis, DNA repair, and chemoresistance [24, 25, 27, 55, 84–89]. It should be emphasized that while depletion of DFS70/LEDGF in cultured cancer cells via knockdown or knockout does not necessarily result in massive cell death, it may impair some of the cancer-associated properties of this protein, particularly in cells under a stressful microenvironment (e.g. presence of cytotoxic drugs). This is likely due to disruption of protein–protein interactions within transcription complexes leading to decreased transcription of stress protective genes and other cancer-associated genes that are critical for tumor progression and therapy resistance. The emerging role of DFS70/LEDGF in cancer, particularly in therapy resistance, makes this protein attractive for therapeutic targeting in combination with other standard cancer treatment modalities.

### Normal functions and regulation

Most studies on the biology of DFS70/LEDGF have been conducted using transformed or cancer cell lines, often under stressful microenvironmental conditions. Thus, we know very little about its biological role(s), cellular/tissue expression, and regulation in non-disease conditions. Early studies established that DFS70/LEDGF is ubiquitously but differentially expressed in human normal tissues, with highest expression in the thymus, heart, brain, skeletal muscle, and ovary [7, 9]. Disruption of the *Psip1* gene, which encodes DFS70/LEDGF, resulting in deletion of the C terminus of this protein in mice was found to cause craniofacial and skeletal malformations that were associated with altered *HOX* gene expression [90]. Although this gene disruption was not intrinsically lethal, the newborn mice died of starvation due to their inability to nurse, which was likely related to structural abnormalities in the olfactory system. A recent chromatin immunoprecipitation sequencing (ChIP-seq) analysis of mouse embryonic stem cells (mESC) with CRISPR/Cas9 knockout of DFS70/LEDGF did not yield a large number of genes affected; however, when the mESC cells were differentiated to embryoid bodies, the knockout affected a substantial number of genes [48]. Taken together these studies implicate DFS70/LEDGF in normal development and cellular differentiation, possibly through a mechanism that involves the reorganization of this and other proteins (e.g. HRP2/HDGF2) with histone modifications to maintain chromatin in a transcriptionally competent state in particular cell types [48].

The transcriptional activity of DFS70/LEDGF is known to be repressed by several factors, including SUMOylation at specific residues, transforming growth factor-β (TGF-β), signal transducer and activation of transcription 3 β (STAT3β), the B-cell lymphoma 2 (Bcl-2) oncogene, and specific microRNAs (e.g. miR-155 and miR-135b) in different contexts (reviewed in Ref. [70]). On the other hand, the transcription factor Sp1, implicated in cancer cell growth and metastasis, is known to bind the DFS70/LEDGF promoter to activate its expression in various cell types (reviewed in Ref. [70]). In addition, the human papilloma virus (HPV) oncoproteins E6 and E7 stimulate oxidative stress and DFS70/LEDGF expression in HPV-positive cervical cancer [85]. As demonstrated recently by our group, DFS70/LEDGF is also upregulated by androgens and glucocorticoids in PCa cells, and downregulated by knockdown of the glucocorticoid receptor, suggesting its susceptibility to nuclear receptor signaling in cancer cells [91].

## Historical perspectives on the autoantibodies to DFS70/LEDGF: what we know

### Detection and clinical associations of anti-DFS autoantibodies

Following the initial discovery of anti-DFS70/LEDGF autoantibodies by Tan’s group, several other groups reported the presence of these antibodies, albeit at various frequencies and titers, in a broad spectrum of inflammatory and miscellaneous conditions. These included atopic diseases, alopecia areata, ocular diseases, chronic fatigue syndrome, arthralgia, fibromyalgia, interstitial cystitis, Behcet’s disease, PCa, healthy individuals, SARD, autoimmune thyroiditis, and others (reviewed in Refs. [70, 92–95]). It is now well established that the anti-DFS70/LEDGF antibodies are primarily IgG (although IgE antibodies were detected in some atopic diseases), recognize a large immunodominant region with discontinuous epitope components encompassing residues 349–435, can be detected at moderate to high titers, and are commonly present, albeit at variable frequencies, in routine ANA cohorts [6, 69, 94–100].

The report by Muro et al. in 2004 that anti-DFS70/LEDGF autoantibodies were present at a frequency of 10.7%, with a broad titer range, in apparently healthy individuals (HI) was the preamble to several studies from laboratories around the world reporting elevated frequencies of these antibodies in HI compared to patients with SARD [101–107]. It has become evident recently that this elevated frequency of anti-DFS70/LEDGF antibodies in HI relative to SARD is influenced by multiple factors, including geography, gender, age, detection assays, and care setting [108–110]. Furthermore, when taking into consideration multiple studies across different countries and institutions, the frequencies of these antibodies in HI and SARD may not appear to be significantly different [70, 92, 95]. However, a distinctive feature of these antibodies in HI individuals is their relatively elevated frequencies as monospecific antibodies (only ANA detectable in the serum by HEp-2 IIFA), in contrast to patients with SARD in which these antibodies tend to appear concomitantly with other disease-associated ANAs [111–113]. These observations have led to the hypothesis that monospecific anti-DFS70/LEDGF autoantibodies may be considered as negative biomarkers to exclude a SARD diagnosis [100–107, 110–115].

A limitation of studies linking anti-DFS70/LEDGF autoantibodies to specific disease conditions, particularly those conducted during the early years of the discovery and characterization of these antibodies, is the lack of very high concordance between the various antibody detection methods used by different laboratories around the world. Many early studies relied mostly on the HEp-2 IIFA test for the detection of these antibodies, with few studies confirming their presence by immunoblotting or enzyme-linked immunosorbent assay (ELISA). This led to high inter-laboratory variability in the reported frequencies of these antibodies (range 0–70%) in different conditions (reviewed in Refs. [70, 92, 95]). These variations have been attributed to confusion of the DFS IIF-ANA pattern with other patterns, different antibody detection substrates and platforms, and often the lack of specific confirmatory assays to support a positive anti-DFS70/LEDGF antibody identification [95, 112–123]. Fortunately, a plethora of new assays for the detection of these autoantibodies have been developed in recent years, providing excellent platforms for the confirmation of their presence in serum with high specificity and sensitivity (reviewed in Ref. [124]). These assays include the NOVA Lite^®^ HEP-2 Select^®^ (Inova Diagnostics), a variation of the HEp-2 IIFA test using immunoadsorption of sera against recombinant DFS70/LEDGF protein; the HEp-2 Elite (Immco Diagnostics), which consist of HEp-2 slides in which the majority of the cells have been depleted of DFS70/LEDGF; the QUANTA Flash^®^DFS70 (Inova Diagnostics), a chemiluminescence immunoassay (CIA) that detects autoantibodies to the IBD containing autoepitope region; ELISA kits containing recombinant DFS70/LEDGF (Euroimmun anti-DFS70/LEDGF ELISA and MBL LEDGF ELISA Kit); line immunoassays including recombinant autoantigens (Euroline ANA Profile 3 + DFS70 LIA^®^, Euroimmun), and dot blot assays (ANA + DFS70 IgG Dot, Alphadia).

Several recent reviews have discussed in detail the current state of knowledge of anti-DFS70/LEDGF autoantibodies [70, 92, 93, 124]. Therefore, we will focus the remaining of this review on discussing briefly several pressing questions that in our opinion need to be addressed thoroughly as the field moves towards uncovering the significance of these antibodies.

### How do we reconcile the reported inter-laboratory differences in the frequencies of anti-DFS70/LEDGF autoantibodies in different disease conditions?

Given the current availability of multiple assay platforms to detect these antibodies there is a need to re-examine more critically, using various methods, their prevalence in large patient cohorts with the atopic diseases, inflammatory conditions, or malignancies in which they were initially detected at variable frequencies. For instance, the frequencies ranging from 10.3 to 71.4% of these antibodies reported in early studies in patients with atopic diseases, using the HEp-2 IIFA test, immunoblotting, and ELISA, have not been corroborated in more recent studies with atopic dermatitis cohorts using the CIA platform [70, 92, 102]. Similarly, the relatively high frequencies (> 25%) detected in early studies with patients with Vogt-Koyanagi-Harada syndrome, Behcet’s disease, sympathetic ophthalmia, and sarcoidosis need to be re-examined in larger patient cohorts using other modern assay platforms [125]. However, to effectively accomplish this goal, efforts should be directed at standardizing in several international centers assay platforms capable of detecting these antibodies with high precision, sensitivity, and inter-laboratory reproducibility. This is particularly critical given the imperfect concordance between the different detection methods, which is likely due to the nature of the DFS70/LEDGF antigen (i.e. full vs truncated recombinant protein vs intracellular protein); antigen source (i.e., bacterial vs eukaryotic derived recombinant protein, which may influence post-translational modification status); assay sensitivity; and inter-laboratory differences in assay performance, data interpretation, and expertise level [95, 116–123]. The availability of a recently developed and well-characterized anti-DFS70/LEDGF autoantibody reference pool will be helpful as a positive control that can be used in the harmonization of different immunoassays for the detection of these autoantibodies [126].

### Are monospecific anti-DFS70/LEDGF autoantibodies clinically reliable biomarkers to rule out a diagnosis of systemic rheumatic disease?

As mentioned above, while the prevalence of anti-DFS70/LEDGF antibodies seems to be comparable in both HI and patients with SARD, typically ranging from 0 to 11% in both groups [70, 92, 95], they differ in their exclusivity, with monospecific antibodies more prevalent in HI than in patients with SARD [100–107, 110–115]. However, monospecific anti-DFS70/LEDGF antibodies are not completely absent in SARD, since a recent international multicenter study led by Marvin Fritzler found a 1.1% frequency of these monospecific antibodies in a large cohort of patients with systemic lupus erythematosus (SLE) (13 out of 1137 patients) [127]. Given the rarity of these monospecific antibodies in SLE, these investigators argued against using them as a criterion for classification or diagnostic purposes [127]. It remains to be determined, however, if the monospecific anti-DFS70/LEDGF antibodies that are rarely detected in SARD patients represent a subset of patients with milder disease or patients with other non-SARD-related inflammatory conditions. Thus, reaching a definite consensus on their utilization as clinically reliable biomarkers for the exclusion of an SARD diagnosis will require additional multicenter trials with large, racially, ethnically and geographically diverse cohorts of different SARDs and healthy controls. These studies should use multiple highly sensitive and specific antibody detection assays, and carefully evaluate available or newly designed ANA-testing algorithms that include these antibodies [97, 103, 104, 128–132].

### How critical it is to confirm the presence of anti-DFS70/LEDGF antibodies in patients with low probability of SARD in a clinical setting?

It has been recognized that the accurate recognition by clinicians of monospecific anti-DFS70/LEDGF antibodies, confirmed by other methods, will reassure patients and their families that a SARD diagnosis is unlikely, which would prevent unnecessary further testing, treatment, and distress to the patient [97, 105, 112–115, 127–129]. Recent studies have provided evidence that the introduction of an anti-DFS70/LEDGF autoantibody test in the clinic not only reduces unnecessary follow-up diagnostic procedures but also minimizes the use of costly health resources generated by suspicion of SARD [128, 129]. However, other clinicians have recently argued against anti-DFS70/LEDGF confirmatory tests on the basis that these antibodies have little clinical benefit, and that ordering instead specific SARD-related autoantibody tests is therefore more beneficial in the clinical setting [133, 134]. Given the documented confusion of the DFS IIF pattern with other SARD-associated ANA patterns and their reported low frequency as monospecific antibodies in SARD [111–113], it is imperative for the rheumatology and clinical immunology community to reach consensus on how critical it is to increase awareness among physicians and clinical laboratory personnel about the importance of correctly identifying and reporting this pattern in a clinical context.

### Do all sera presenting a monospecific DFS immunofluorescence pattern target DFS70/LEDGF?

Support for the specificity of the DFS IIF pattern comes from several studies indicating that when this pattern is accurately identified, the presence of DFS70/LEDGF autoantibodies is typically confirmed by complementary methods in a majority of the sera [95, 119–123, 135, 136]. In a previous study we addressed this issue by evaluating 64 human sera that presented a monospecific DFS IIF pattern for the presence of antibodies to DFS70/LEDGF or its interacting partner MeCP2, a protein of approximately 70 kD that also displayed the DFS IIF pattern in HEp-2 cells and in advanced prostate cancer cells, which express high levels of both DFS70/LEDGF and MeCP2 [135]. The presence of autoantibodies to DFS70/LEDGF was confirmed by CIA, immunoblotting of cancer cell lysates with and without depletion of this protein, and immunoabsorption experiments using an IBD containing autoepitope peptide [135]. Interestingly, we observed that 61 of the 64 DFS70 sera reacted with DFS70/LEDGF in the multiple assays, with high concordance between the assays, and none appeared to react against MeCP2. Three of the sera with a putative DFS IIF pattern were negative by CIA and also showed very weak or negative reactivity by immunoblotting. Our follow-up study with a Mexican patient population also yielded high concordance between different methods to detect anti-DFS70/LEDGF antibodies [136].

These results led us to conclude that sera presenting a monospecific DFS IIF pattern are highly specific for antibodies against DFS70/LEDGF, although we cannot exclude conclusively the possibility that there is a minority of monospecific DFS-positive sera that display negative immunoreactivity for this protein using methods other than HEp-2 IIFA test. Indeed, there have been several studies in which the presence of antibodies to DFS70/LEDGF in certain sera presenting a monospecific DFS IIF pattern has not been confirmed by immunoblotting or other methods. For instance, in their initial characterization of the DFS70/LEDGF autoantibodies, Ochs et al. [6] reported that out of 18 sera with the DFS IIF pattern, 4 did not react with the recombinant protein by immunoblotting in spite of 2 sera having relatively high titers (≥ 320). Other investigators, including our group, have also reported discrepancies between the HEp-2 IIFA test results and other confirmatory assays in a fraction of sera presenting the DFS IIF pattern [69, 79, 120, 122]. This issue was underscored in a study by Bizzaro et al. [120] demonstrating that about 50% of serum samples with a positive DFS IIF pattern scored positive by at least one of several CIA or immunoblot methods evaluated in the study. Given that all the methods evaluated included a DFS70/LEDGF antigen that contained the IBD autoepitope region, these investigators concluded that there is no difference in the overall diagnostic accuracy among methods that use truncated or full-length DFS70/LEDGF protein, and that other antibodies may be responsible for producing DFS70-like pattern at the HEp-2 IIFA test.

**Fig. 6:**
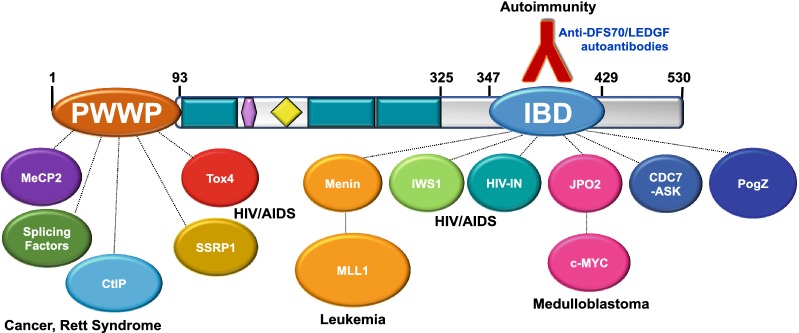
The DFS70/LEDGF interactome. The PWWP domain is the site for several protein–protein interactions that include MeCP2, splicing factors, and others. Several proteins expressed in mammalian cells, and overexpressed in cancer cells, interact directly with the IBD, a region that also corresponds to the autoepitope targeted by anti-DFS autoantibodies. These proteins may form complexes with other proteins that come into close proximity to DFS70/LEDGF such as c-Myc and MLL1 to facilitate their tethering to transcriptionally active sites within chromatin. Some of these interactions have already been implicated in various human pathologies, including HIV/AIDS, Rett syndrome, cancer, and leukemia

It is possible that a subset of anti-DFS70/LEDGF autoantibodies may recognize conformational epitopes that do not renature in some immunoassays, or post-translationally modified (PTM) amino acids in the IBD region of DFS70/LEDGF that are not present in the recombinant protein used in some assays, especially if not derived from a eukaryotic expression system. This could explain the presence of serum autoantibodies producing “DFS-like’ or “pseudo-DFS” IIF patterns in HEp-2 cells but that display negative immunoreactivity for anti-DFS70/LEDGF autoantibodies using other methods, which would account for the inter-assay discrepancies in the detection of these antibodies observed by several groups [137]. On the other hand, it is also conceivable that while DFS70/LEDGF is the primary target of sera presenting the DFS IIF pattern, a small fraction of these sera may contain autoantibodies to other proteins that are part of nuclear complexes involving this protein. These target proteins may include interacting partners of DFS70/LEDGF that co-localize with this protein in active chromatin, some of which have molecular weights around 65–75 kD, such as MeCP2, CDC7/ASK, and Menin (Table 2, Fig. 6). Other interacting partners, such as MLL (431 kD) and PC4 (17 kD), migrate with very high or low molecular weights, and are difficult to detect by conventional immunoblotting of cell lysates. In addition, we have observed in preliminary studies that some interacting partners of DFS70/LEDGF have differential expression (low vs high) in cancer cells depending on the context (i.e. chemosensitivity, chemoresistance, tumor stage, etc.), which would make them difficult to detect by immunoblotting in some cell lysate preparations (unpublished results). It is also possible that the HRP2/HDGF2 protein, which works in concert with DFS70/LEDGF to facilitate RNA pol II transcription and has an IBD region [48, 53, 54], may also be the target of some sera presenting the DFS IIF pattern. Thus, we cannot rule out the possibility that sera presenting this pattern contain antibodies to specific DFS70/LEDGF interacting partners or other components of the RNA pol II transcription complex and active chromatin. This possibility, which are currently exploring, awaits experimental verification.

### Are autoantibodies to DFS70/LEDGF present with increased frequency in patients with specific cancers?

This question has not been fully addressed since there are very few reports exploring the presence of these antibodies in specific cancer patient cohorts. As mentioned above, our research on the role of DFS70/LEDGF in cancer was triggered by the observation that PCa patients who came to Loma Linda University Medical Center for proton therapy exhibited a relatively elevated frequency of antibodies presenting the DFS IIF pattern in HEp-2 cells (14%, 29 out of 207 patients) compared to matched controls (4.2%) and blood bank donors (2.3%) [79]. Confirmatory studies showed that 18% of the PCa patients reacted positively with a full-length recombinant DFS70/LEDGF by ELISA, using a stringent cut-off, but not all the positive sera reacted strongly with this protein by immunoblotting using whole lysates of PCa cells [79]. The increased reactivity of these and other PCa sera against DFS70/LEDGF in ELISA was confirmed in a subsequent independent study [138]. Interestingly, several PCa sera presenting the DFS IIF pattern also showed moderate to strong reactivity against the presumed DFS70/LEDGF protein band (75 kD) in cell lysates but gave negative results by ELISA [79]. At the time we interpreted these discrepancies as resulting from differences in the assay platforms or nature of the DFS70/LEDGF antigen used (e.g. native protein in fixed cancer cells vs bacterial-derived recombinant protein vs denatured protein in cell lysates). However, at present we cannot rule the possibility that some of these antibodies were targeting interacting partners of DFS70/LEDGF with similar migration in immunoblots that may have been concomitantly upregulated with this protein in the PCa cells.

Consistent with these observations, two other independent groups confirmed the presence of autoantibodies to DFS70/LEDGF in PCa patients, although the frequencies were not reported. In one study, Xie et al. [139] demonstrated that the inclusion of this protein in a panel of 6 tumor-associated antigens for autoantibody detection using a multiplex assay, enhanced sensitivity and specificity in distinguishing PCa cases (n = 141) from non-malignant cases (n = 250). O’Rourke et al. [140] using a native antigen reverse capture microarray for autoantibody profiling in a PCa cohort (n = 41) showed that DFS70/LEDGF (identified as PSIP1 in that study) was one of the top 5 autoantibody signatures in this cohort, and that these signatures differentiate PCa from other malignancies. In contrast, an IIF-ANA survey of anti-DFS70/LEDGF autoantibodies, conducted by Bizzaro et al. [141] in 334 cancer patients representing 27 different malignancies, revealed a low frequency of these antibodies (1.8% overall in cancer, 2% in 48 PCa). In agreement with these results, Mahler et al. [102], using CIA, were unable to detect any anti-DFS70/LEDGF autoantibodies in 40 patients with diverse cancer types. Given the overexpression of DFS70/LEDGF and its interactions with multiple partners in different types of cancer cells it would be reasonable to expect that this protein becomes the target of a cancer-associated autoantibody response in a subset of patients. Nevertheless, to date the studies on the presence of anti-DFS70/LEDGF antibodies in cancer are inconclusive, which would merit additional studies using larger, geographically and racially diverse cancer cohorts and multiple complementary antibody-detection assays.

### What factors trigger the autoantibody response to DFS70/LEDGF, particularly to its IBD region?

The fact that the immunodominant region targeted by anti-DFS70/LEDGF autoantibodies overlaps almost perfectly with the IBD region recognized by HIV-IN may at first glance suggest a link between these antibodies and exposure to the HIV-1 virus [142]. We know that during HIV-1 infection, HIV-IN binds to the IBD and uses DFS70/LEDGF to tether the viral genome to active chromatin as a mechanism to ensure efficient viral integration and replication, and that during virus assembly the integrase keeps this protein associated with the viral particle, most likely to provide an integration advantage to newly released virions [143]. It could be speculated that the DFS70/LEDGF complex with HIV-IN may alter the immunological processing of this protein, leading to the generation of an antibody response to the IBD region in HIV-infected individuals. However, this does not seem to be the case as Shoenfeld et al. [144] recently demonstrated a 0% frequency of these antibodies in HIV-infected patients. However, it remains to be determined if these antibodies are capable of binding to DFS70/LEDGF ensnared within circulating viral particles, and are present in HIV-resistant individuals, potentially serving a protective role. Notably, a lab-generated VH antibody domain that binds the DFS70/LEDGF IBD region interfered with HIV-IN interaction with this domain and viral infectivity in vitro, raising the prospect that antibodies that mimic HIV-IN by targeting the IBD region, such as anti-DFS70/LEDGF autoantibodies, could be used to intracellularly immunize T cells in HIV-positive patients [145].

The immunological targeting of the IBD region of DFS70/LEDGF, a critical functional domain, is consistent with the established notion that autoantibodies target highly conserved and functionally important structural domains [1]. As mentioned above, DFS70/LEDGF’s intrinsically disordered structure, its interactions with multiple proteins through the IBD, and the generation during apoptosis of cleavage fragments of this protein retaining the IBD, may make this highly conserved domain more prone to become the target of a humoral response in genetically susceptible individuals. This response could be amplified in the presence of specific environmental or physiological factors, or inflammatory conditions, that may upregulate DFS70/LEDGF expression in specific tissues. Based on our knowledge of the biology of DFS70/LEDGF, there are several factors that produce an augmented state of cellular oxidative stress and trigger its overexpression, including malignant transformation, HPV infection, increased androgen- and glucocorticoid-receptor signaling, and exposure to certain environmental stressors (e.g. cytotoxic drugs, UV irradiation, alcohol) that elevate intracellular ROS levels [15–24, 70, 82–85, 91]. Interestingly, during increased oxidative stress, DFS70/LEDGF undergoes thioredoxin 1-mediated PTMs consisting of cysteine reductions [146]. It is not clear, however, if these or any other types of stress-associated PTMs enhance the immunogenicity of this protein, which would be consistent with evidence pointing to a role of oxidative stress-associated PTMs in forming neoepitopes in autoantigens that give rise to ANAs and other autoantibodies [147]. Thus, it is conceivable that the autoantibody response to DFS70/LEDGF might be a “sensor” of increased oxidative stress associated with the upregulation and possibly post-translational modification of this protein under a pro-inflammatory microenvironment.

### Are anti-DFS70/LEDGF autoantibodies protective?

This question raises the interesting possibility, reviewed recently by Infantino et al. [148], that these autoantibodies may serve a protective role and could be exploited for therapeutic purposes. However, to date the evidence supporting this possibility is limited and largely circumstantial. As Infantino et al. argued, the notion that these autoantibodies may play a protective role is based on a few indirect observations: (1) follow-up studies averaging 4 to 10 years show that HI with monospecific anti-DFS70/LEDGF antibodies rarely develop SARD or other chronic diseases [92, 97, 105, 112]; (2) these monospecific antibodies are rarely found in SLE patients and when present may not be associated with musculoskeletal activity or the presence of anti-B2 glycoprotein 1 antibodies, [127]; (3) three patients with dermatomyositis complicated with interstitial lung disease showed increased levels of anti-DFS70/LEDGF antibodies, with concomitant decrease of disease-marker anti-MDA5 antibodies, as they went into remission after treatment, whereas one patient that failed treatment and died from the disease showed unchanged levels of anti-MDA5 antibodies and disappearance of anti-DFS70/LEDGF antibodies [149]; (4) anti-DFS70/LEDGF antibodies were found at a higher frequency in patients with undifferentiated connective tissue disease (UCTD) compared to patients with connective tissue disease, which could potentially help identify UCTD patients who will not progress to SARD [150]; and (5) unpublished data showing that 40% of NZBx/F1 female mice immunized weekly with affinity purified anti-DFS70/LEDGF autoantibodies had minor lupus nephritis compared to controls [148].

It is evident from these observations that while the prospects of a protective effect of these antibodies is very attractive and plausible, additional well-controlled studies with human cohorts, as well as animal and cellular models are urgently needed to establish this protective role. In a previous review, we discussed how the protective effect of these antibodies could be context-dependent, raising the possibility that they may also behave as cytotoxic antibodies under certain conditions [70]. This latter function is supported by a few early studies showing that anti-DFS70/LEDGF antibodies are cytotoxic to cultured lens epithelial cells and lens organs, possibly by blocking extracellularly released DFS70/LEDGF from re-entering cells, thus preventing it from protecting cells against microenvironmental stressors [151, 152]. If this cytotoxic function occurs in vivo, it would be consistent with the reported elevated frequency of these autoantibodies in patients with cataracts and other ocular diseases (reviewed in Refs. [10, 16, 70, 92]), which should be confirmed using a combination of current detection assays. It should be noted that the extracellular secretion of DFS70/LEDGF has only been reported in the context of cultured LECs overexpressing this protein tagged with green fluorescent protein (GFP) [8]. Singh et al. [8] demonstrated that GFP-LEDGF overexpressing LECs cultured under thermal and oxidative stress not only were more resistant to stress-induced cell death but also secreted the fluorescently tagged protein into the culture medium. When anti-DFS70/LEDGF antibodies (lab generated) were added to the medium there was a decreased accumulation of fluorescent DFS70/LEDGF in the nuclei of cultured cells that was associated with increased cell death [8]. The cytotoxic effects of autoantibodies to DFS70/LEDGF and the extracellular release of this protein, if confirmed in other contexts, could be exploited for therapeutic interventions in diseases where this protein plays a pathological role, such as HIV/AIDS and cancer.

## Conclusions

Compelling evidence points to a biological role for DFS70/LEDGF as a DNA-associated protein that interacts with multiple partners to tether them to transcriptionally active sites in chromatin as a mechanism to modulate RNA pol II transcription, mRNA splicing, and DNA repair. Because of these fundamental functions, DFS70/LEDGF is able to contribute to several biological processes, including development, cellular stress response and survival, malignant transformation, and HIV-1 integration. Some of the protein–protein interactions involving this protein have already been implicated in various pathologies, including HIV/AIDS, medulloblastoma, leukemia, prostate cancer, Rett Syndrome, and autoantibody responses (Fig. 6).

As we commemorate this year the 20th anniversary of the first report on the characterization of the DFS70/LEDGF autoantibody/autoantigen system, we are still in search for answers to our old question of “what exactly are the anti-DFS70 autoantibodies trying to tell us?” [4]. While it is undisputable that the past 20 years have brought a better understanding of the basic biology of DFS70/LEDGF in health and disease, and more clarity about the clinical utility of the anti-DFS70/LEDGF antibodies as potential biomarkers for the exclusion of an SARD diagnosis, it is also evident that the clinical and biological significance of these autoantibodies still remain enigmatic. A careful consideration of the most pressing questions in the field, some of which were briefly discussed above, should advance research aimed at unraveling the significance and potential clinical and therapeutic utility of these antibodies. An in-depth knowledge of the biology of DFS70/LEDGF and the clinical significance of its associated autoantibodies promises to yield translational innovations that could dramatically improve the lives of patients with diseases in which this protein is aberrantly regulated and functionally relevant.

## Data Availability

The datasets used and/or analyzed during the current study are available from the corresponding author on reasonable request.

## Abbreviations

ADH: alcohol dehydrogenase
AIDS: acquired immunodeficiency syndrome
ALB: albumin
ALDH: aldehyde dehydrogenase
ANA: antinuclear autoantibodies
AOP2/PRDX6: antioxidant protein 2/peroxiredoxin-6
AARD: ANA-associated rheumatic diseases
ATH: adenine-thymine hook
Bcl-2: B cell lymphoma 2
CDC-ASK: subunit and activator of S-phase kinase
cDNA: complementary deoxyribonucleic acid
ChIP-seq: chromatin immunoprecipitation sequencing (ChIP-seq)
CIA: chemiluminescence immunoassay
CR: charged region
CRISPR/Cas9: clustered regularly interspaced short palindromic repeats/Cas 9 enzyme
CtIP: carboxy-terminal interacting protein
CYGB: cytoglobin
DFS: dense fine speckles
DFS70: dense fine speckled protein of 70 kilodaltons
DNA: deoxyribonucleic acid
ELISA: enzyme-linked immunosorbent assay
ERp57: endoplasmic reticulum protein of 57 kilodaltons
FACT: facilitates chromatin transcription
FBXO10: F-box only protein 10
FxFG: phenylalanine–glycine repeats
GFP: green fluorescent protein
HATH: homologous to amino terminus of hepatoma-derived growth factor
H3K36me2/3: dimethylated and trimethylated lysine 36 in histone H3
HDGF: hepatoma derived growth factor
HI: healthy individual
HIV-IN: human immunodeficiency virus 1 integrase
HOX: homeobox
HPV: human papilloma virus
HRP2/HDGF2: hepatoma derived growth factor related protein 2
HSP27: heat-shock protein of 27 kilodaltons
IBD: integrase binding domain
IBM: IBD-binding short linear motif
ICAP: International Consensus on ANA Patterns
IDR: intrinsically disordered region
IgG: immunoglobulin G
IgE: immunoglobulin E
IIF: indirect immunofluorescence
IIFA: indirect immunofluorescence assay
IL-6: interleukin-6
INV: involucrin
IWS1: interacts with SUPT6H, carboxy-terminal domain assembly factor 1
JPO2/CDCA7L: cell division cycle-associated 7-like protein
kD: kilodaltons
LEC: lens epithelial cell
LEDGF/p75: lens epithelium-derived growth factor protein of 75 kilodaltons
LIA: line immunoassay
MDA5: melanoma differentiation-associated gene 5
MeCP2: methyl-CpG-binding protein 2
MEN1: menin 1
mESC: mouse embryonic stem cells
MHC II: major histocompatibility complex II
MLL: mixed leukemia lineage
mRNA: messenger ribonucleic acid
NLS: nuclear localization signal
NOVA1: RNA-binding neuro-oncological ventral antigen 1
NUP98: nucleoprotein 98
p21: protein 21
PC4: positive coactivator 4
PCa: prostate cancer
PIP3-E/IPCEPF-1: interactor protein for cytohesin exchange factors 1
PogZ: pogo transposable element with zinc finger domain
PSIP1: PC4 and SFRS1 interacting protein 1
PTM: post-translational modification
PWWP: proline-tryptophan-tryptophan-proline
RNA: ribonucleic acid
RNA pol II: RNA polymerase 2
RTT: Rett syndrome
SARD: systemic autoimmune rheumatic diseases
SLE: systemic lupus erythematosus
siRNA: small interference RNA
shRNA: short hairpin RNA
SOD3: extracellular superoxide dismutase
SRD: supercoiled recognition domain
SRSF1: serine/arginine-rich splicing factor 1
SSRP1: structure specific recognition protein 1
STAT3β: signal transducer and activation of transcription 3 beta
TALEN: transcription activator-like effector nuclease
TGF-β: transforming growth factor beta
TOX4: toxoplasmosis high mobility group box family member 4
TPO: thyroid peroxidase
UCTD: undifferentiated connective tissue disease
UV: ultraviolet light
VEGF-C: vascular endothelial growth factor C
αB-crystallin: alpha beta crystallin
ΔC: deletion in carboxy-terminal domain
ΔN: deletion in amino-terminal domain
γGCS-HS: gamma glutamyl cysteine synthase heavy subunit
